# Progress toward an integrated understanding of Parkinson’s disease

**DOI:** 10.12688/f1000research.11820.1

**Published:** 2017-07-12

**Authors:** Maxime W.C. Rousseaux, Joshua M. Shulman, Joseph Jankovic

**Affiliations:** 1 Jan and Dan Duncan Neurological Research Institute, Texas Children’s Hospital, 1250 Moursund St, Houston, TX, 77030, USA; 2 Department of Molecular and Human Genetics, Baylor College of Medicine, One Baylor Plaza, Houston, TX, 77030, USA; 3 Parkinson's Disease Center and Movement Disorders Clinic, Department of Neurology, Baylor College of Medicine, 7200 Cambridge, Houston, TX, 77030-4202, USA; 4 Department of Neuroscience, Baylor College of Medicine, One Baylor Plaza, Houston, TX, 77030, USA

**Keywords:** Parkinson's disease, PD, neurodegenerative disorders, α-Synuclein, Parkinson's disease genetics

## Abstract

Parkinson’s disease (PD) is the second most common neurodegenerative disorder after Alzheimer’s disease, affecting over 10 million individuals worldwide. While numerous effective symptomatic treatments are currently available, no curative or disease-modifying therapies exist. An integrated, comprehensive understanding of PD pathogenic mechanisms will likely address this unmet clinical need. Here, we highlight recent progress in PD research with an emphasis on promising translational findings, including (i) advances in our understanding of disease susceptibility, (ii) improved knowledge of cellular dysfunction, and (iii) insights into mechanisms of spread and propagation of PD pathology. We emphasize connections between these previously disparate strands of PD research and the development of an emerging systems-level understanding that will enable the next generation of PD therapeutics.

## Introduction

Parkinson’s disease (PD) is the most common movement disorder, affecting 2–3% of individuals over the age of 65 ^1^. It is clinically characterized by a core set of motor manifestations, including tremor, slow movement (bradykinesia), increased muscle tone (rigidity), and gait and postural impairment as well as a variety of other motor and non-motor features, including cognitive impairment, depression, pain and other sensory symptoms, autonomic dysfunction, and others ^2^. PD is characterized pathologically by the loss of predominantly dopaminergic neurons, associated with intracellular, insoluble α-synuclein (α-Syn) aggregates, largely localized to cytoplasmic inclusions termed Lewy bodies and within neuronal processes termed Lewy neurites. Whereas current treatments can ameliorate the cardinal motor symptoms, no disease-modifying therapies exist ^3^. A large body of research has therefore focused on understanding the biological mechanisms that underlie disease onset and progression, with the goal of developing effective pathogenesis-targeted, disease-modifying therapies.

As this year marks the 200-year anniversary of the recognition of PD by James Parkinson ^4^, we also celebrate the remarkable progress in understanding PD etiology and pathogenesis ^5^. Here, we review recent advances in the study of the genetics, cell biology, and pathology of PD, highlighting emerging areas of overlap. Where these areas were previously studied in isolation, results from these disparate strands of research are beginning to converge, providing a more unified understanding of PD pathogenesis. We argue that this integrative approach to PD, in which seemingly disconnected results are re-examined as components of a cohesive whole, is also creating exciting opportunities for clinical applications.

## Functional genomics and the promise of personalized medicine

Although only a small minority of patients with PD have thus far been found to have responsible pathogenic gene mutations, genetic discoveries have nevertheless been a driving force in the elucidation of PD mechanisms. Following on the early success from studies of rare families with Mendelian forms of PD, more than 20 PD genes and variants have now been implicated, including from large-scale genome-wide association studies (GWAS) and, more recently, whole exome sequencing (WES) studies in population-based cohorts ^6– 8^. A common challenge with either approach entails definitive confirmation of the responsible genes and elucidation of implicated disease mechanisms. In GWAS, implicated genomic regions often contain several equally plausible gene candidates. Whereas WES studies usually single out specific gene candidates, the implicated alleles may be too rare to definitively confirm a causal link to PD based on currently available sample sizes. Medium- to high-throughput screening assays in cellular or animal models can connect promising candidate genes to PD-relevant biologic mechanisms, prioritizing a subset for further study. For example, Jansen *et al.* ^9^ evaluated 27 promising candidate genes with homozygous or compound heterozygous loss-of-function alleles based on WES in 1,148 unrelated young-onset PD cases. Since nearly all of the gene candidates were observed only once in the cohort, the investigators probed each gene for roles in mitochondrial dynamics or α-Syn-mediated toxicity using cellular and fruit fly experimental models. Ultimately, five genes were supported by both functional data and additional human genetic analyses consistent with replication. Beyond accelerating the discovery of novel PD genes, related approaches are also revealing the function of many other established genes/variants, grouping discrete susceptibility loci into common pathways and thereby consolidating our understanding of PD pathogenesis. For example, the recently identified PD gene *CHCHD2* may mediate its activity through a mitochondrial pathway like other recessive PD genes (see below) ^10– 12^. Moreover, *VPS35* and *EIF4G1*, both implicated in autosomal dominant forms of PD ^13– 17^, were recently found to genetically interact with one another and converge on α-Syn toxicity in yeast, worm, and mouse models of synucleinopathy ^18^.

By contrast, with the sequence-based discovery of rare variant PD risk factors, the susceptibility alleles identified by GWAS ^7^ usually do not alter protein-coding regions, making functional follow-up more challenging. For example, one of the earliest discovered PD risk polymorphisms at the human *MAPT* locus may primarily impact alternative mRNA splicing ^19, 20^. In another recent study, Soldner *et al.* used human pluripotent cell-derived neurons containing an intronic PD-related variant in the gene encoding α-Syn ( *SNCA*) ^21^. The authors found that this common polymorphism, which is present in about half of the population, coincided with a distal enhancer element resulting in an approximately 10% increase in *SNCA* transcript levels. It was therefore suggested that a mild increase of α-Syn over the course of decades renders individuals susceptible to PD. This is consistent with the findings from rare families with *SNCA* locus multiplication. In these cases, individuals with *SNCA* gene mulptiplication present with clinical features typical of PD, including a similar age at onset to sporadic PD ^22, 23^, whereas individuals with *SNCA* gene triplication present with a more early onset, aggressive form of PD ^24^. Thus, *SNCA* gene dosage may be an integral feature of PD pathogenesis. In the case of the more common *SNCA* polymorphism, the modest increase in α-Syn protein levels may interact with other genetic risk variants or environmental exposures to cause PD. For example, Goldman *et al.* found that head injury was significantly associated with increased PD risk, but only in the context of a disease-associated *SNCA* promoter polymorphism ^25^.

One of the great hopes for advances in PD genetics is to realize goals for personalized medicine, including improved risk prediction and even targeted therapies. It has long been speculated that much of PD’s clinical heterogeneity may be genetically encoded ^26, 27^. For example, besides their potent impact on risk of PD ^28^, *GBA* mutations have been reported by several groups to cause an earlier age-at-onset, more rapid progression, and an increased risk of cognitive impairment and dementia in carriers ^29– 34^. Moreover, additional studies have looked at the effect of allelic heterogeneity on modifying PD clinical presentations ^35– 37^. In the future, it may be important to couple such studies examining the clinical impact of selected allelic variants with experimental investigations to define functional consequences in well-defined cellular or animal models. Moreover, once characterized, these models can serve as a platform for testing putative “personalized” treatments ^38– 42^. For example, Sanofi Genzyme is currently supporting a study (MOVES-PD) of GZ/SAR402671, a glucosylceramide synthase inhibitor, in PD patients carrying a *GBA* gene mutation (ClinicalTrials.gov, NCT02906020). Another study (AiM-PD) is examining the effects of oral Ambroxol, a glucocerebrosidase-modulating chaperone, in patients with PD (ClinicalTrials.gov, NCT02941822). Lastly, the use of biomarkers in stratifying clinical populations and understanding the biological underpinning of PD subtypes will be critically important when developing personalized medicine approaches. Specifically, profiling blood and CSF biomarkers may enhance disease subtyping based on clinical manifestations alone. This hypothesis is currently being tested in the Parkinson’s Progression Markers Initiative (PPMI) led by the Michael J. Fox Foundation for Parkinson’s Research ^43, 44^.

## From genes to organelles and cellular homeostasis

The maintenance and function of cellular organelles, including mitochondria and lysosomes, are critical for functional neuronal integrity ^45– 47^. Interestingly, several previously identified PD genes such as *PARK2*, *PARK6*, and *PARK7* (encoding Parkin, PTEN-induced putative kinase 1 [PINK1], and DJ-1, respectively) have been linked to mitochondrial function ^48– 54^. Since their initial discovery, a large body of work has elucidated a cellular pathway through which dysfunctional mitochondria can be recycled by way of autophagy or, more specifically, “mitophagy” ^55– 59^. Damaged mitochondria promote the phosphorylation of ubiquitin and Parkin by PINK1 and are subsequently degraded by the autophagosomal system ^59– 62^. Additionally, studies of these genes have provided insight into their mitochondrial functions in healthy and diseased contexts. For example, a recent study suggests that Parkin acts as an endogenous buffer for mitochondrial stress and its loss sensitizes dopaminergic neurons to mitochondrial mutations over time ^63, 64^. In addition, using a fly model, Vos and colleagues ^65^ discovered a new pathway that could suppress the motor and biochemical abnormalities caused by *PINK1* loss of function. Specifically, PINK1 genetically interacts with the enzyme responsible for the conversion of vitamin K1 into vitamin K2 ^65^. Remarkably, supplementation of vitamin K2 could reverse *PINK1* mutant phenotypes, suggesting a potential therapeutic approach. However, patient selection will be critical for potential clinical trials, as *PARK6* mutations result in rare, autosomal recessive juvenile parkinsonism ^66^, and it is uncertain whether a similar functional deficiency in vitamin K2 may apply in the general PD patient population. Identifying the subcellular impact of other PD genes may similarly lead to other targeted therapies.

Beyond discovering the primary targets of genetic abnormalities, it is essential to understand the subsequent cascade of cellular injury, such as how damaged mitochondria impact other cellular constituents, leading to neuronal dysfunction and death. In other words, discrete targets must be understood in the context of a cohesive, dynamic system. One example comes from recent studies that strongly implicate defects in the vesicular trafficking system ^67^, which mediates cellular secretion and endocytosis as well as vesicle docking and fusion and is critically important for synaptic transmission, lysosomal degradation, and autophagy. Several PD genes, including *VPS35*, *LRRK2*, *RAB7L1*, *GBA*, and *SNCA*, have functions that converge on the vesicular trafficking system ^67– 70^. Leucine-rich repeat kinase 2 (LRRK2) and Rab-7-like protein 1 (RAB7L1), for example, act coordinately to regulate endolysosomal protein sorting via Rab GTPases, and several studies also support a key role for the cargo-shuttling retromer protein vacuolar protein sorting 35 (VPS35) ^71– 73^. In fact, the retromer is implicated as a critical downstream effector whose dysfunction may lead to neuronal toxicity and death ^74^. Importantly, VPS35 may also play a role in mitophagy, perhaps via trafficking of mitochondria-derived vesicles to lysosomes ^75^. The dense interconnections between PD genes and other regulators of vesicular trafficking were highlighted by recent work that combined an α-Syn protein–protein interaction network with suppressor-enhancer screening in yeast ^76, 77^. Based on these and other findings, chemical modulators of autophagy and/or the retromer, such as rapamycin (or similar “rapalogues”) ^78, 79^ and R55 ^80, 81^, respectively, may be promising therapeutic avenues for targeting vesicular sorting defects in PD. However, since these pathways mediate essential functions in most tissues, successful dose titration to achieve selective action in the nervous system while minimizing potentially deleterious off-target effects is one anticipated challenge ^82^. Nevertheless, these studies illustrate how the emerging, systems-based understanding of PD can highlight vulnerable “nodes” within complex cellular networks, creating promising therapeutic opportunities.

## α-Syn toxicity and propagation: from cells to systems

The centrality of α-Syn in PD pathogenesis was established nearly two decades ago with the dual finding that (i) this protein is the principal constituent of the hallmark Lewy body pathology ^83^ and (ii) *SNCA* gene mutations cause familial forms of PD ^84^. Since then, α-Syn genomic locus multiplication ^22– 24^ or promoter polymorphisms that increase protein expression ^21^ have also been confirmed as causal factors. Thus, intensive investigation has probed the relationship between α-Syn with PD pathogenesis. α-Syn was first described as a member of the synuclein family, which is associated with the synapse and the nucleus ^85^. While studies on the physiological function of α-Syn suggest that it may play a role in synaptic transmission ^86^ and aid in curving cellular membranes ^87^, less is known regarding the mechanism(s) through which its gain-of-function causes neurodegeneration. Thus, one aspect of α-Syn research has focused on understanding how this protein causes toxicity within the cell. Studies in both animal models and human tissue have highlighted a role for α-Syn at the outer mitochondrial membrane ^88, 89^, the nucleus ^90– 92^, and the synapse ^93, 94^ as putative toxic mechanisms. Moreover, mechanisms involving proteostasis ^95, 96^ and lysosomal dysfunction ^45^ that collectively lead to increased α-Syn levels are also tantalizing.

Given the direct relationship between α-Syn abundance and its role in PD pathogenesis and propagation, immunotherapy against α-Syn has emerged as one of the most promising therapeutic approaches for PD ^97, 98^. Results from ongoing and future immunotherapeutic trials by Prothena/Roche, Biogen, AFFiRiS, and other biotech companies will provide information on whether aggregated α-Syn is an important therapeutic target ^99^. Nevertheless, caution is warranted given previous negative outcomes from immunotherapeutic trials of other neurodegenerative diseases, such as Alzheimer’s disease, characterized by protein aggregation (proteinopathies) ^100^.

Another avenue for therapeutic intervention that is actively being investigated is based on the observation that hyperactivity of the non-receptor tyrosine kinase c-Abl contributes to α-Syn phosphorylation, accumulation, and neurodegeneration ^101^. This has led to preliminary investigation of Nilotinib, a c-Abl inhibitor previously approved for the treatment of chronic myeloid leukemia, as a potential therapeutic agent for PD ^102^. Further studies, however, are needed before this potent drug can be recommended as a symptomatic or disease-modifying therapy for PD ^103^. A single-center trial at Georgetown University (ClinicalTrials.gov, NCT02954978) and a multicenter trial (NILO-PD) (ClinicalTrials.gov, NCT02281474), sponsored by the Michael J. Fox Foundation for Parkinson’s Research and the Parkinson Study Group, are currently under way.

Since the seminal observation by Braak and colleagues that most idiopathic PD cases fit a relatively predictable, caudal to rostral pathologic staging progression ^104^, there has been great interest in understanding the mechanism by which α-Syn pathology may spread from the peripheral organs (e.g. the enteric or pericardial tissue) via the vagus nerve to the lower brainstem and eventually involve the neocortex. The intriguing possibility that α-Syn pathology can spread from cell to cell ^105– 107^ was suggested by observations of Lewy-like pathology in engrafted neurons from PD patients receiving fetal dopaminergic cell transplants ^108, 109^. First studies indicated that aggregates of α-Syn could enter cells that express transgenic α-Syn and seed the formation of new aggregates ^110, 111^. More recently, research findings have indicated that even synthetic, wild-type forms of α-Syn, if improperly folded and injected in the mouse brain, can induce the misfolding of otherwise normal endogenous α-Syn, thereby amplifying and propagating pathological forms ^112– 114^, properties consistent with those of prions (infectious proteinaceous agents) ^115^. It appears that α-Syn can adopt a variety of different misfolded/aggregated oligomeric conformations that correspond to distinct profiles of toxicity in experimental assays ^116, 117^. These findings raise the intriguing, though yet unproven, hypothesis that certain α-Syn “strains” may contribute to clinical and pathologic heterogeneity among PD and other synucleinopathies. Interestingly, Mao *et al.* ^118^ recently identified lymphocyte-activation gene 3 (LAG3) as a candidate receptor for α-Syn oligomeric “seeds”, and genetic manipulation of LAG3 in cells and mouse models altered pathologic progression. These findings raise the possibility of diagnostic and therapeutic advance based on the detection of α-Syn strains in patient populations as well as potential pharmacologic blockade of propagation ^118^. It is also possible that neuroanatomic variation among key co-factors or receptors for α-Syn seeding or spread might contribute to the selective vulnerability of specific neuronal subpopulations in PD. Despite remarkable recent progress, the clinical relevance of α-Syn seeding and propagation (if any) remains to be fully understood ^105, 119, 120^. For example, autopsies from some PD patients receiving fetal grafts were devoid of pathology two decades following transplantation ^121, 122^. Another recent study found that individuals receiving human growth hormone derived from human cadavers were at no greater risk of developing PD ^123^. The extent of pathological spread of α-Syn does not always follow a defined trans-synaptic pattern nor does the brain areas it affects fully correlate with clinical measures ^105^. Thus, a continued investigation into the mechanism through which α-Syn pathological assemblies form and how these are tied to toxicity is warranted.

Gastrointestinal dysmotility is a common early complaint in PD patients ^124, 125^, and the enteric nervous system has been implicated as an early target of α-Syn pathology ^104, 126^. Along with the growing interest in immunologic and inflammatory disease mechanisms, the gut microbiome has recently come under investigation in studies of both PD patients ^127– 130^ and animal models ^131^. Sampson and colleagues ^131^ found that α-Syn transgenic mice living in a germ-free environment were less vulnerable to neurodegeneration, but when the mice were inoculated with fecal bacteria taken from patients with PD their motor function deteriorated. While this study suggests the possibility that the gut microbiome may influence PD manifestations, it will be important to define the specific microbial contributors to the disease as well as recapitulate these findings in other disease models before moving forward into humans ^132^. Beyond its potential role in disease modulation, the gastrointestinal tract might even be a trigger point for disease based on the findings of early enteric nervous system α-Syn pathology in some pathologic series ^104^. Long before the current excitement concerning α-Syn propagation, Braak *et al.* speculated about a possible gastrointestinal pathogen (or other enteric exposure) as an initiating event, followed by pathologic spread via the vagus nerve to its medullary dorsal motor nucleus where PD changes first appear in the brainstem ^104, 126, 133^. Following up on this provocative hypothesis, investigators recently found that subjects undergoing vagotomy (transection of the vagal nerve for the treatment of peptic ulcer disease) are at a modest but significantly reduced risk of PD ^134, 135^. While intriguing, the finding requires further confirmation, and animal model studies will be essential to definitively prove that the mechanism of protection is indeed based on the disruption of spread from the enteric to the central nervous system.

## Conclusion

**Figure 1.**
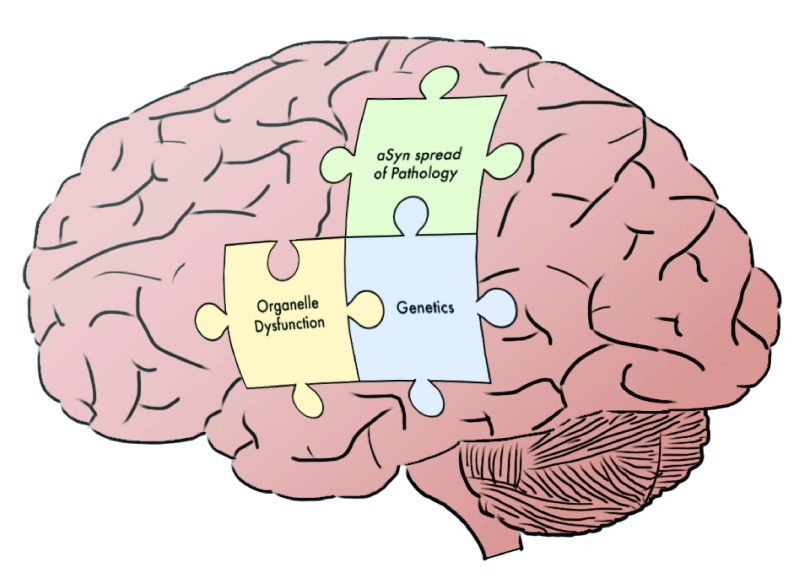
Putting the Parkinson’s disease (PD) puzzle together. Three pieces of the “PD puzzle” covered in this short review are highlighted within the context of the brain. Additional elements remain to be fully elucidated. The integrated, systems-level understanding of PD that emerges from understanding how these components fit together will likely accelerate therapeutic advances. α-Syn, α-synuclein.

Recent advances have clearly enhanced our knowledge of the fundamental processes underlying PD pathogenesis. As connections are recognized among the disparate domains of PD inquiry, the broader patterns begin to emerge. As discussed above, we now recognize the cellular targets of many PD genes, such as how *PARKIN* and *GBA* regulate mitochondrial or lysosomal function, respectively. Additionally, the field has made progress toward understanding how dynamic interactions between such organelles impact overall cellular health, as in mitophagy. Lastly, studies of α-Syn illustrate the convergence of classical histopathologic analysis of PD with genetic investigations, and more recent investigations demonstrate how synucleinopathy not only impacts single cells or tissues but also may propagate throughout the nervous system. In sum, we are rapidly making progress toward a more cohesive model of PD pathogenesis, and this systems-level understanding is likely to accelerate therapeutic inroads. With the broad outlines of the “PD puzzle” now apparent ( Figure 1), we predict that new insights can be more rapidly integrated within this framework. For example, forthcoming discoveries of new genetic risk loci can be understood within the context of known functional pathways within both neurons and other cell types, such as astrocytes or microglia. An integrated understanding of PD will also enable more effective multi- and inter-disciplinary collaboration among scientists and clinicians, driving next-generation therapeutic trials targeting disease mechanisms and fulfilling the promise of personalized medicine. Advances in understanding the cellular mechanism underlying PD-related neurodegeneration will undoubtedly lead to better symptomatic and novel pathogenesis-targeted, disease-modifying therapies ^136^.

## Abbreviations

α-Syn, α-synuclein; GWAS, genome-wide association study; LAG3, lymphocyte-activation gene 3; PD, Parkinson’s disease; PINK1, PTEN-induced putative kinase 1; VPS35, vacuolar protein sorting 35; WES, whole exome sequencing.
